# Autistic traits and social anxiety predict differential performance on social cognitive tasks in typically developing young adults

**DOI:** 10.1371/journal.pone.0195239

**Published:** 2018-03-29

**Authors:** Cheryl L. Dickter, Joshua A. Burk, Katarina Fleckenstein, C. Teal Kozikowski

**Affiliations:** 1 Psychological Sciences, College of William & Mary, Williamsburg, VA, United States of America; 2 Psychiatry & Behavioral Sciences, Eastern Virginia Medical School, Norfolk, VA, United States of America; Brigham Young University, UNITED STATES

## Abstract

The current work examined the unique contribution that autistic traits and social anxiety have on tasks examining attention and emotion processing. In Study 1, 119 typically-developing college students completed a flanker task assessing the control of attention to target faces and away from distracting faces during emotion identification. In Study 2, 208 typically-developing college students performed a visual search task which required identification of whether a series of 8 or 16 emotional faces depicted the same or different emotions. Participants with more self-reported autistic traits performed more slowly on the flanker task in Study 1 than those with fewer autistic traits when stimuli depicted complex emotions. In Study 2, participants higher in social anxiety performed less accurately on trials showing all complex faces; participants with autistic traits showed no differences. These studies suggest that traits related to autism and to social anxiety differentially impact social cognitive processing.

## Introduction

Navigating everyday social situations is crucial for human survival and necessitates a range of social cognitive processes in which individuals must monitor social context and meanings along with others’ behaviors [1]. Individuals with autism spectrum disorder (ASD) typically exhibit impairments in many social cognitive processes which can lead to impaired social interactions [2]. In particular, some persons with ASD experience deficits in emotion identification and attentional control compared with individuals not on the autism spectrum (e.g., [3,4]), although they tend to perform better in tasks requiring attention to detail (e.g., [5]). Social interactions may be accompanied by social anxiety, which can also affect the social cognitive processes involved in communication. As trait social anxiety is often co-morbid with ASD, research is needed to examine how traits related to autism and to social anxiety differentially affect social cognitive processes related to communication. The current set of studies aimed to do so with the goal of better understanding the unique contributions of traits related to autism and to social anxiety in tasks involving the processing of social stimuli and that allowed manipulation of attentional demands.

Many individuals with autism are thought to have impairments in the recognition and interpretation of facial expressions [6]. Emotion identification is a complex process in which individuals have to employ a configural processing strategy to detect changes in the relationships between facial features that communicate specific emotions [7–9]. Some studies have shown that individuals with ASD tend to fixate on particular facial features such as the mouth, particularly with dynamic images, rather than processing the relative position of facial features as a global image [10–12]. A meta-analysis by Uljarevic and Hamilton [13] demonstrated that deficits in emotion identification in individuals with ASD are robust (but see [14]).

Emotion identification in individuals with ASD also seems to depend on the complexity of the emotion. Although there is evidence that those with ASD do not show deficits in the identification of basic emotions like happiness or anger [15], they have difficulties recognizing more complex emotions such as surprise ([16,17], but see [18]). As facial emotional expressions are an important source of information about others’ emotional states [19] and intentions [20], it is important to further investigate whether emotion processing is affected in individuals with autistic traits. Deficits in detecting emotional expressions could lead to challenging social interactions, affecting a range of life outcomes, including education level achieved and employment status [21].

Studies have shown that individuals with autism often demonstrate superior processing, including performing better on a range of visuo-spatial tasks involving non-social stimuli that require attention to details, compared with neurotypical individuals [5]. An enhanced discrimination ability has been reported in individuals with ASD in tasks using neutral stimuli such as letters. For example, children [22] and adults [5] with ASD are less affected by the similarity of target and distractor letters in cognitive tasks than matched control groups [23–25]. A meta-analysis has supported the idea that individuals with ASD tend to have differences in processing that can lead to complicated patterns across tasks, including superiority on some tasks and impairments on others [26]. This meta-analysis also reported considerable heterogeneity within samples. Using tasks that allow for the investigation of responses to social stimuli such as faces permit the opportunity to test whether the advantages in visual search tasks are maintained when faces depicting different emotions are presented. This issue is important because individuals often need to navigate scenarios involving multiple people in the social world.

In tasks that require attention to multiple faces, unlike tasks that use relatively neutral stimuli, persons with ASD show reduced attention to emotional expressions [27,28] and perform worse than those without ASD [29,30]. The discrepancy between performance in non-social and social tasks may be explained by work by Dichter and Belger [31]. They demonstrated that although similar brain regions were activated in participants with and without ASD on a task assessing attention to non-social stimuli (i.e., the arrow flanker task), the cognitive control network was activated less in participants with ASD compared to control participants when social stimuli (i.e., gaze stimuli) were presented.

Taken together, the findings described above suggest that individuals with autism tend to have impaired emotion identification, impaired attentional control, and superior attention to detail compared to individuals without autism. This body of work has focused on comparing individuals with autism to those without autism. Researchers have suggested that, due to the nature of autism as a spectrum disorder, studying the Broad Autism Phenotype (BAP; [32]) can lead to a stronger understanding of the mechanisms underlying ASD [33]. The BAP is a set of challenges with communication, social skills, and cognitive processing similar to deficits associated with autism, but not as severe as in diagnosed individuals [34]. The BAP was originally conceptualized based upon observations of unusual behaviors in parents of children with autism, but has been expanded to reflect that everybody in the general population falls along the BAP continuum [35,36]. The BAP has been largely ignored in the study of emotion identification and attentional processing, despite the fact that autistic traits are distributed and heritable in the general population [37–39]. The research that has investigated this population has shown that individuals with high levels of autistic traits, but without a diagnosis of ASD, demonstrate impairments in attention to faces [40–42] and configural face processing [43,44]. Thus, studying individuals along the BAP may be useful for gaining insight into processing differences that lay along a continuum involving diagnosed and non-diagnosed individuals.

The BAP is also associated with differences in social anxiety, which is characterized by fear and avoidance of social situations [2]. Individuals with a greater number of autistic traits report higher levels of social anxiety [32,33]. This is consistent with research suggesting that social anxiety is often comorbid with ASD, with estimates ranging from a 49% [45] to 57% [46] co-occurrence in young adults. Research has failed to find consistent associations between social anxiety and emotion identification in typically developing individuals (see [47]). However, in individuals with ASD, social anxiety contributes to impaired facial emotion recognition through reduced fixation on the eyes [48] and deficits in attending to facial features [2]. The type of emotion is also important in the relationship between social anxiety and emotion identification. That is, socially anxious individuals are impaired in understanding complex emotions [49], although less research has examined emotion identification. Socially anxious individuals also tend to show differences in processing emotions based on valence, such that they demonstrate more amygdala activation from negative than positive emotions [50], which may lead to greater attention to negative emotions. The social components of ASD and the symptoms of social anxiety are correlated yet distinguishable constructs that yield unique electrophysiological activation during social cognitive tasks [51] and each construct contributes unique variance to the success of certain social interventions [52].

In the current studies, we employed two tasks that have been used to assess attention and modified them to include social stimuli in order to examine face processing. In Study 1, we adapted Eriksen’s flanker task [53] to assess the control of attention in a task in which participants must identify an emotion depicted on a face presented among distracting ‘flanker’ faces depicting the same or a different emotion. Reaction times were used to measure how well participants were able to control their attention away from flanker faces and correctly identify the target emotion. Study 2 employed a modified visual search paradigm in which a number of faces were displayed simultaneously on a computer screen and participants indicated whether or not all of the faces depicted the same emotion. Accuracy in the visual search task is a measure of the ability to attend to details that distinguish emotions. For both tasks, we varied the complexity (i.e., how much cognition is involved in the processing of the emotion, or how subtle the emotion is) and valence (i.e., positive or negative) of the faces presented in both studies with four emotions varying by both dimensions: angry (basic, negative), fear (complex, negative), happy (basic, positive), and surprise (complex, positive) [16,29,54,55].

The goal of the current work was to examine the unique contribution of autistic traits and social anxiety to performance in these social cognitive tasks. Autistic traits were measured using the Autism Spectrum Quotient (AQ), a self-report measure that quantifies an individual’s level of autistic traits [56]. Social anxiety was assessed with the Social Phobia and Anxiety Inventory (SPAI-23; [57]). Because individuals who self-report high levels of autistic traits generally show elevated levels of social anxiety [58–60], we assessed these traits separately to examine differences in performance on the two tasks.

We predicted that performance would differ as a function of the task as well as individual traits. These results were expected based on the nature of the tasks. In a task that involves the identification of an emotion (i.e., flanker task), there is a large body of literature showing that individuals who report high levels of autistic traits (high AQ) show impairments, particularly with complex emotions [16,29,54,55]. Therefore, on the flanker task, we expected that high AQ, but not low AQ, participants would perform slowly on trials involving complex emotions. Although some research suggests that social anxiety may be associated with a decreased ability to understand complex versus basic emotions, this research has not examined emotion identification in faces. Thus, it was unclear whether the SPAI would affect performance in the flanker task.

On the visual search task, which involves attention to detail, we expected no differences between high and low AQ participants. High AQ individuals may be less accurate at identifying complex and negative emotions than low AQ individuals, so they can use their superior attention to detail skills to discriminate between faces [10–12]. Because social anxiety is associated with a decreased ability to recognize complex versus basic emotions [49]), we predicted that high anxiety participants may be less accurate in trials when the targets and distractors are complex. This may be exacerbated by trials with more faces, as socially anxious individuals tend to view faces depicting emotions as threatening, which can lead to an attentional bias away from faces [61]; indeed, individuals diagnosed with social anxiety disorder were more distracted when numerous threatening words were presented in a visual search task [62]. To investigate this possibility, we manipulated the number of faces displayed (i.e., 8 or 16) and predicted that high anxiety participants would be less accurate on trials depicting faces with complex emotions but only on trials with a large number (i.e., 16) of faces. Regarding valence, fMRI research has demonstrated that socially anxious participants show increased amygdala activation to emotions with negative valence compared to positive valence [50]. This suggests that greater attention to negative emotions may lead high SPAI participants to have a higher accuracy identifying target faces depicting negative emotions when the distractors are positive.

## Study 1 method

### Participants

Undergraduate students (N = 119; 53 males; *M*_*age*_ = 19.3 (*SD* = 1.26) years; 70.2% White, 4.8% Multiracial, 6.7% Black, 6.7% Hispanic, 9.6% Asian, 1.9% other) from a medium-sized university in the southeastern United States participated for either course credit in an introductory psychology class or monetary remuneration. All procedures were approved by the Protection of Human Subjects Committee and informed consent was acquired electronically. To determine our sample size, power analyses were conducted at .80 power, α = .05, with two between-subjects groups. This analysis indicated that the minimum sample size needed for a medium effect size is 51 participants. Because we planned to only examine the responses of participants in the top and bottom third of our two individual trait measures and thus expected to exclude one third of our sample, we oversampled by recruiting approximately 100 participants.

### Materials

#### Flanker task

**Fig 1:**
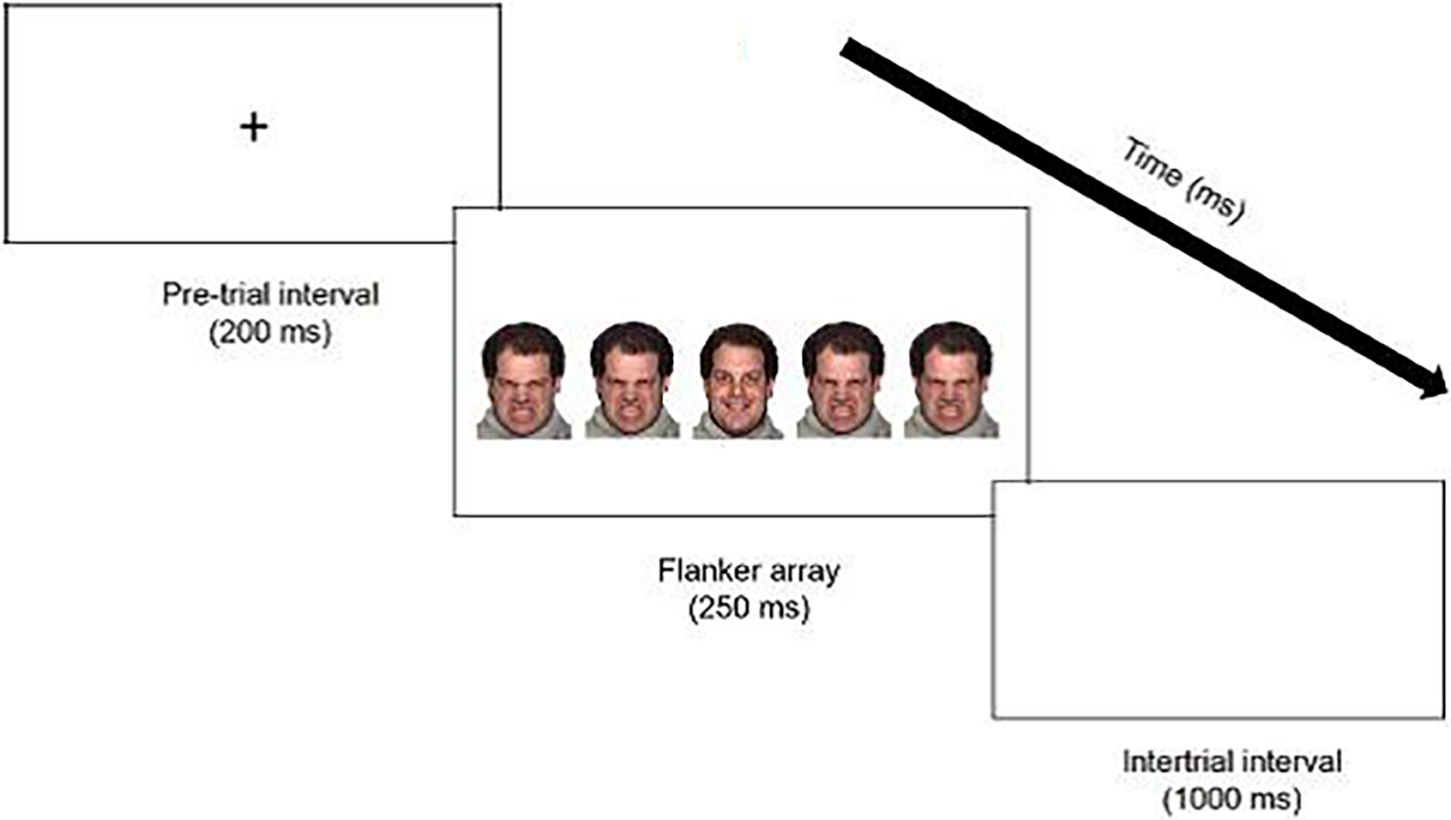
Example of flanker task trial.

Color images of White male faces from the NimStim Set of Facial Expressions were used as stimuli [63]. The pictures included the same models displaying basic emotions (i.e., happy, angry) and complex emotions (i.e., surprise, fear) [19]. Fig 1 depicts an example trial. Stimuli were presented in 5-picture arrays. Each trial consisted of a 200 ms pre-stimulus baseline period followed by a stimulus array in which a centrally-presented target emotion face was flanked by two emotion face stimuli on the left and right sides. The four flanker emotion faces were identical to one another. Arrays were presented for 250 ms with an inter-trial interval of 1000 ms. Participants completed six blocks of 72 trials each. Their task was to identify the emotion on the central (target) face as one of two emotions as quickly as possible by pressing one of two designated computer keys. Two emotions were included for each block; the order of the blocks (i.e., the order of the different emotion combinations) was counterbalanced across participants. The hand assignment for each emotion was counterbalanced across blocks. The probability of compatible and incompatible trials was 50% throughout the study.

#### Autism Quotient

The Autism Quotient questionnaire (AQ) is a 50-item self-report measure of autism-spectrum behaviors [56]. The AQ has been used in previous work to study the BAP (e.g., [64]). Participants respond to statements (e.g., “I find it hard to make new friends”; “I tend to notice details that others do not”) using a 4-point scale (1 = *definitely agree*, 2 = *slightly agree*, 3 = *definitely disagree*, 4 = *slightly disagree*). Dichotomous scoring was used with higher scores indicating more autistic behaviors.

#### Social Phobia and Anxiety Inventory

The Social Phobia and Anxiety Inventory (SPAI-23) is a 23-item self-report measure of social phobia [57] which has strong reliability and validity in college students [65]. Participants rate statements (e.g., “I feel anxious before entering a social situation”) according to the following five-point Likert-type scale: 0 = *never*, 1 = *very infrequent*, 2 = *sometimes*, 3 = *very frequent*, and 4 = *always*.

### Procedure

Groups of up to four students participated simultaneously in a university computer lab in which students had their own computer with a privacy screen. After providing informed consent, students completed the flanker task. Both reaction time and accuracy for each trial were recorded. Participants were instructed to sit straight, keep both feet on the floor, and remain as still as possible during the task. After the task was finished, students completed the questionnaires and provided demographic information. Sessions lasted approximately 45 minutes.

## Results

Fifteen participants were excluded for non-compliance (e.g., not following task instructions), computer problems (e.g., software error forced the task to end early; computer crashed) or experimenter errors (e.g., experimenter entered an incorrect participant number in the behavioral task).

### Analytic strategy

In order to compare participants with larger and smaller numbers of autistic traits, two groups were formed based on scores in the top (“high”) and bottom (“low”) third on the total AQ; these means, presented in Table 1, were significantly different from one another, *t*(67) = -17.82, *p* < .001. Five participants in the high AQ group scored at or above the clinical cut-off (32) suggested for this measure [6]. In order to compare participants with more and fewer traits related to social anxiety, two groups were formed based on scores in the top (“high”) and bottom (“low”) third on the SPAI; these means were significantly different from one another, *t*(66) = -5.19, *p* < .001. Twenty participants in the high SPAI group scored at or above the clinical cut-off (30) suggested for this measure [57]. The AQ and the SPAI were significantly correlated with one another, *r* = .46, *p* < .001. The dependent variable for all subsequent analyses was reaction time on correct trials. Two analyses of variance (ANOVAs) were conducted, one comparing basic and complex emotions and one comparing negative and positive emotions. The four emotions were happy (basic, positive), angry (basic, negative), surprise (complex, positive) and fear (complex, negative). Only significant main effects and interactions including AQ or SPAI as factors are reported below, as those findings were of theoretical interest in the present study. Greenhouse-Geisser p-values are reported where appropriate. Effect sizes using partial eta-squared are reported, in which a small effect should yield a value of approximately .01, a medium effect about .09, and a large effect approximately .25 [66].

**Table 1:** Mean and standard deviation (in parentheses) of AQ and SPAI scores.

	High AQ	Low AQ	High SPAI	Low SPAI
Study 1				
	n = 35	n = 34	n = 34	n = 34
	28.23 (3.40)	13.65 (3.39)	24.95 (9.80)	14.18 (6.69)
Study 2				
	n = 50	n = 45	n = 51	n = 63
	27.72 (4.30)	10.73 (2.88)	32.29 (4.83)	9.49 (5.55)

### Autistic behaviors (AQ)

#### Emotion complexity

**Fig 2:**
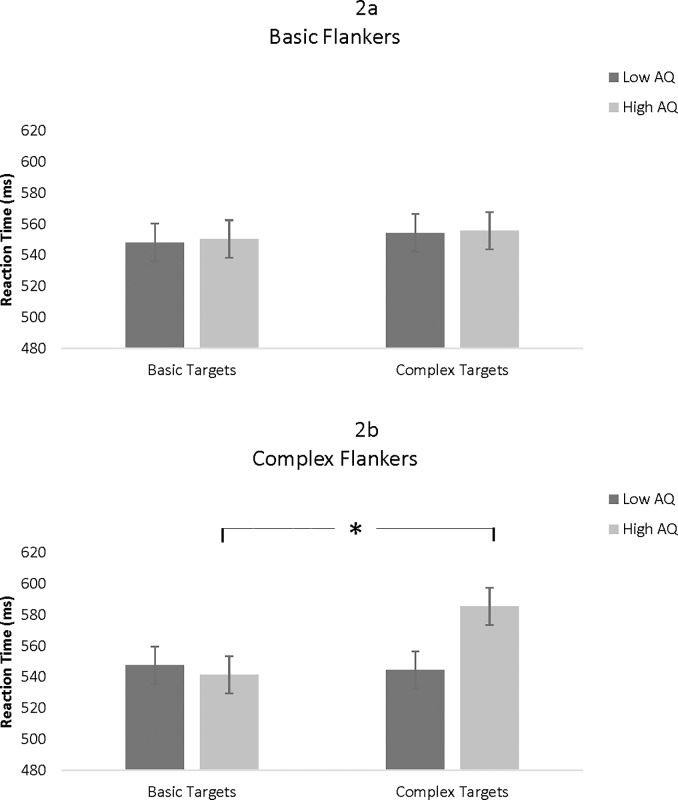
Reaction time as a function of flanker complexity, target complexity, and AQ. Fig 2a depicts basic flankers while Fig 2b shows complex flankers. The asterisk indicates statistical significance at *p* < .05. Error bars represent standard errors.

A 2 (AQ group: high, low) x 2 (target: basic, complex) x 2 (flanker: basic, complex) mixed-factor ANOVA revealed a significant AQ x Target x Flanker interaction, *F*(1, 66) = 6.34, *p* = .014, η_p_^2^ = .088. This interaction was explicated by conducting separate Target x AQ ANOVAs for each flanker type. There were no significant effects for the basic flankers, *F*(1, 66) = 0.07, *p* = .939. For the complex flankers, however, there was a significant Target x AQ interaction, *F*(1, 66) = 7.46, *p* = .008, η_p_^2^ = .102. Simple main effects analyses showed that for the low AQ group, there was no difference when the targets were basic or complex, *F*(1,33) = 0.05, *p* = .831, but with the high AQ group, complex targets yielded slower responses than basic targets when the flankers were complex, *F*(1,33) = 21.25, *p* < .001, η_p_^2^ = .392, as depicted in Fig 2.

#### Emotion valence

A 2 (AQ group: high, low) x 2 (target: positive, negative) x 2 (flanker: positive, negative) mixed-factor ANOVA did not yield a significant AQ x Target x Flanker interaction, *F*(1, 66) = 0.73, *p* = .397.

### Social anxiety (SPAI)

#### Emotion complexity

A 2 (SPAI group: high, low) x 2 (target: basic, complex) x 2 (flanker: basic, complex) mixed-factor ANOVA did not reveal a SPAI x Target x Flanker interaction, *F*(1, 69) = 0.17, *p* = .681.

#### Emotion valence

A 2 (SPAI group: high, low) x 2 (target: positive, negative) x 2 (flanker: positive, negative) mixed-factor ANOVA did not yield a SPAI x Target x Flanker interaction, *F*(1, 69) = 0.02, *p* = .883.

## Study 2

The results of Study 1 demonstrated that individuals with autistic traits were slow to respond on trials involving complex emotional faces on a task in which they identified the emotion presented on a target’s face. The SPAI did not affect task performance. With the flanker task, slower reaction times are indicative of a weaker ability to control attention away from distracting stimuli, which captured the attention of participants with autistic traits when those stimuli were complex emotional faces. High SPAI participants, however, were unaffected by the type of stimuli presented.

To better understand the processing of emotions in those with traits related to autism and social anxiety, Study 2 was designed to assess performance on a visual search task that involves attention to detail, rather than the control of attention, using emotional faces.

## Method

### Participants

Two hundred eight undergraduate students (59.3% women; *M*_*age*_ = 18.9 [*SD* = 1.25 years]; 61% White, 0.5% Native American, 2.9% Pacific Islander, 7.7% Black, 6.3% Hispanic, 9.6% Asian, 12.0% other) from a medium-size liberal arts institution completed this experiment for either course credit in an introductory psychology class or a small monetary compensation. All procedures were approved by the university’s Protection of Human Subjects Committee and informed consent was acquired electronically. Power analyses conducted at .80 power, α = .05, with two between-subjects groups yielded a minimum sample size of 50 participants. As with Study 1, we oversampled to account for our plans to only use data from two-thirds of our sample to examine the responses of participants in the top and bottom third on each of our two individual trait measures.

### Materials

#### Modified visual search task

**Fig 3:**
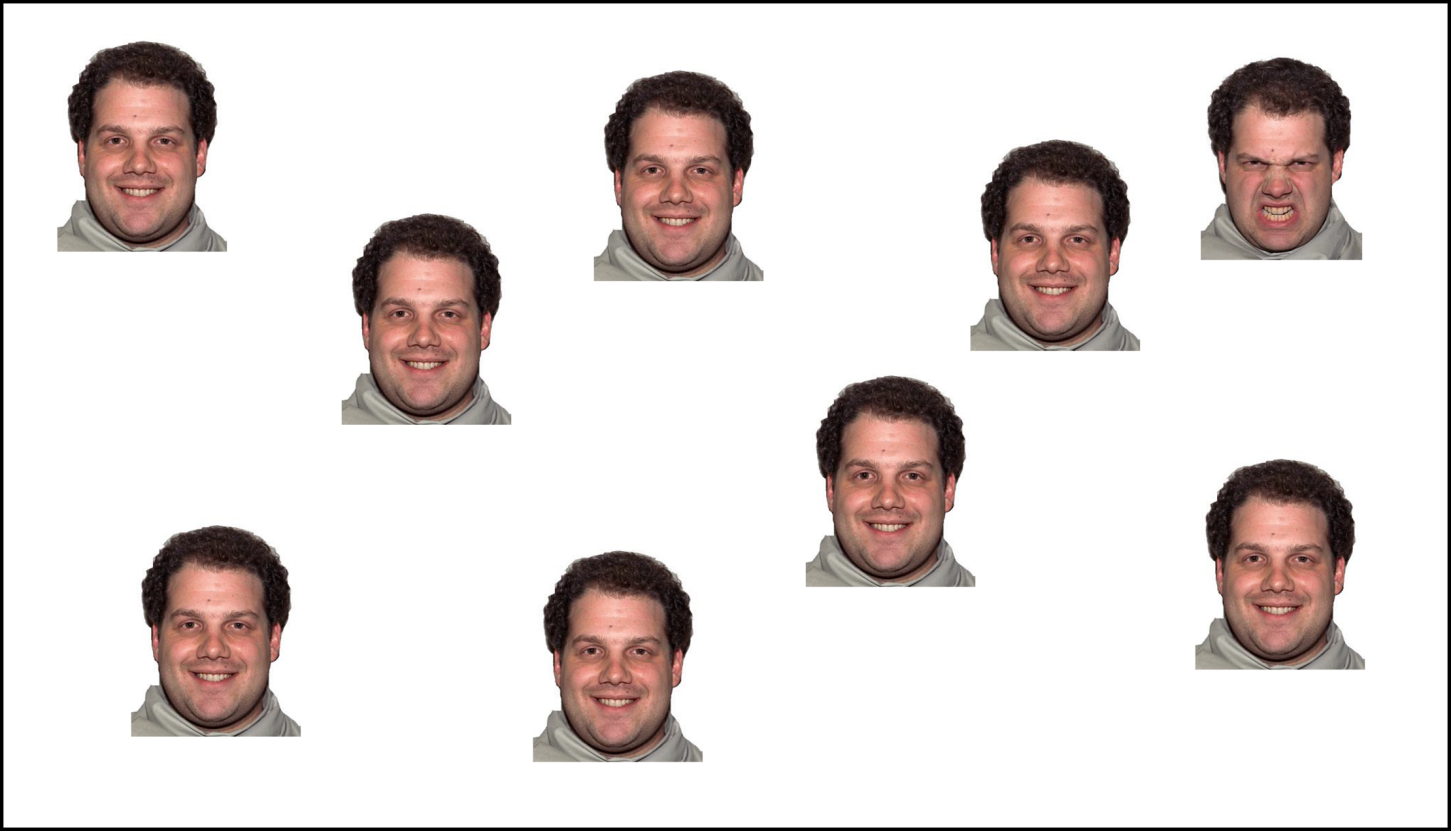
Example of visual search task trial.

The same color image faces used in Study 1 were used in the visual search task. As depicted in Fig 3, the visual search task consisted of a series of trials in which a fixation cross was presented for 200 ms followed by an array of face stimuli that stayed on the screen until the participant responded. Each trial was followed by an intertrial interval of 500 ms. The facial stimuli were presented in an array with one target face embedded in a matrix of other distractor faces. Half of the trials presented eight faces; the other half presented 16 faces. Stimuli were presented around the central point of the screen. The placement of the target face on the screen varied randomly throughout the testing session and none overlapped with one another. There were four types of trials: basic target-basic distractor, basic target-complex distractor, complex target-basic distractor, and complex target-complex distractor. Participants were instructed to press one key if all of the faces on the screen displayed the same emotion and another key if one of the faces displayed a different emotion than the others; the hand assignment was counterbalanced across participants.

#### Autism Quotient

The AQ in this study was identical to that used in Study 1.

#### Social Phobia and Anxiety Inventory

The SPAI-23 used in this study is identical to that used in Study 1.

### Procedure

The study was conducted with groups of up to four students in an on-campus computer lab. Each participant had his or her own computer protected by privacy screens. To limit movement, they were instructed to sit up straight, to keep both feet on the floor, and to remain as still as possible during the task. Participants completed one block of 256 trials, followed by the SPAI, AQ and demographic questionnaires.

## Results

Participants were excluded for non-compliance (n = 3), computer problems (n = 4), experimenter errors (n = 4), and medical issues (broken arm, broken finger, n = 2).

### Analytic strategy

Participants who had an average accuracy score less than 20% were excluded from the analyses. As with Study 1, participants were separated into the top and bottom third based on their AQ score in order to compare participants with larger and smaller numbers of autistic traits. These mean AQ scores, as presented in Table 1, were statistically significantly different from one another, *t*(93) = -22.38, *p* < .001. Eight participants in the high AQ group scored at or above the clinical cut-off (32) suggested for this measure [6]. In order to compare participants with more and fewer traits related to social anxiety, two groups were formed from the top and bottom third of the sample on the SPAI. Mean SPAI scores were statistically significantly different from one another, *t*(112) = -23.10, *p* < .001. Twenty-eight participants in the high SPAI group scored at or above the clinical cut-off (30) suggested for this measure [57]. The AQ and the SPAI were significantly correlated with one another, *r* = .42, *p* < .001. The dependent variable for analyses was accuracy for trials in which there was a discrepant emotion as we were interested in examining participants’ attention and emotion identification of a face among distractor faces depicting different emotions. For AQ and SPAI, two ANOVAs were conducted, one comparing basic and complex emotions and one comparing negative and positive emotions. Greenhouse-Geisser p-values are reported where appropriate. Effect sizes using partial eta-squared are reported, in which a small effect should yield a value of approximately .01, a medium effect about .09, and a large effect approximately .25 [66]. Only significant main effects and interactions including AQ or SPAI as factors are reported below, as those findings were of theoretical interest in the present study.

### Autistic behaviors (AQ)

#### Emotion complexity

**Fig 4:**
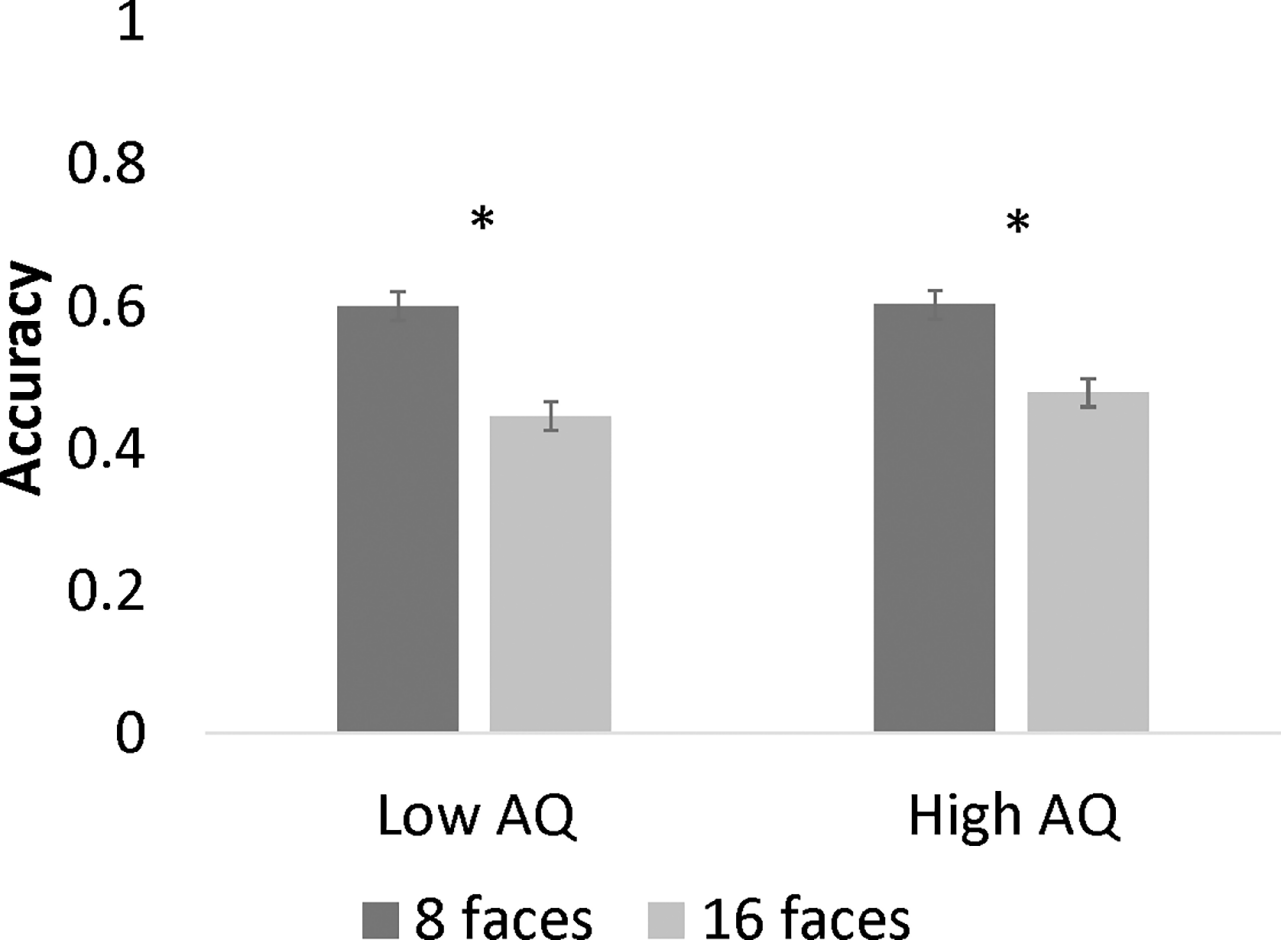
Accuracy as a function of number of stimuli and AQ. The asterisks indicate statistical significance at *p* < .05. Error bars represent standard errors.

A 2 (Number of Stimuli: 8, 16) x 2 (Target: basic, complex) x 2 (Distractor: basic, complex) x 2 (AQ Group: high, low) mixed factor ANOVA revealed a Number of Stimuli x AQ interaction, *F*(1, 93) = 4.21, *p* = .043, *η*_*p*_^*2*^ = .043, as depicted in Fig 4. Simple main effects showed that low AQ participants were more accurate when there were 8 stimuli than 16 stimuli, *F*(1, 44) = 231.85, *p* < .001, *η*_*p*_^*2*^ = .840. High AQ participants were also more accurate when there were 8 stimuli than 16 stimuli, *F*(1, 49) = 130.34, *p* < .001, *η*_*p*_^*2*^ = .727. Thus, both groups of participants showed the same pattern of responses based on emotion complexity when number of stimuli were varied.

**Emotion valence.** A 2 (Number of Stimuli: 8, 16) x 2 (Target: positive, negative) x 2 (Distractor: positive, negative) x 2 (AQ Group: high, low) mixed factor ANOVA did not yield any main effects or interactions that included AQ as a factor.

### Social anxiety (SPAI)

#### Emotion complexity

**Fig 5:**
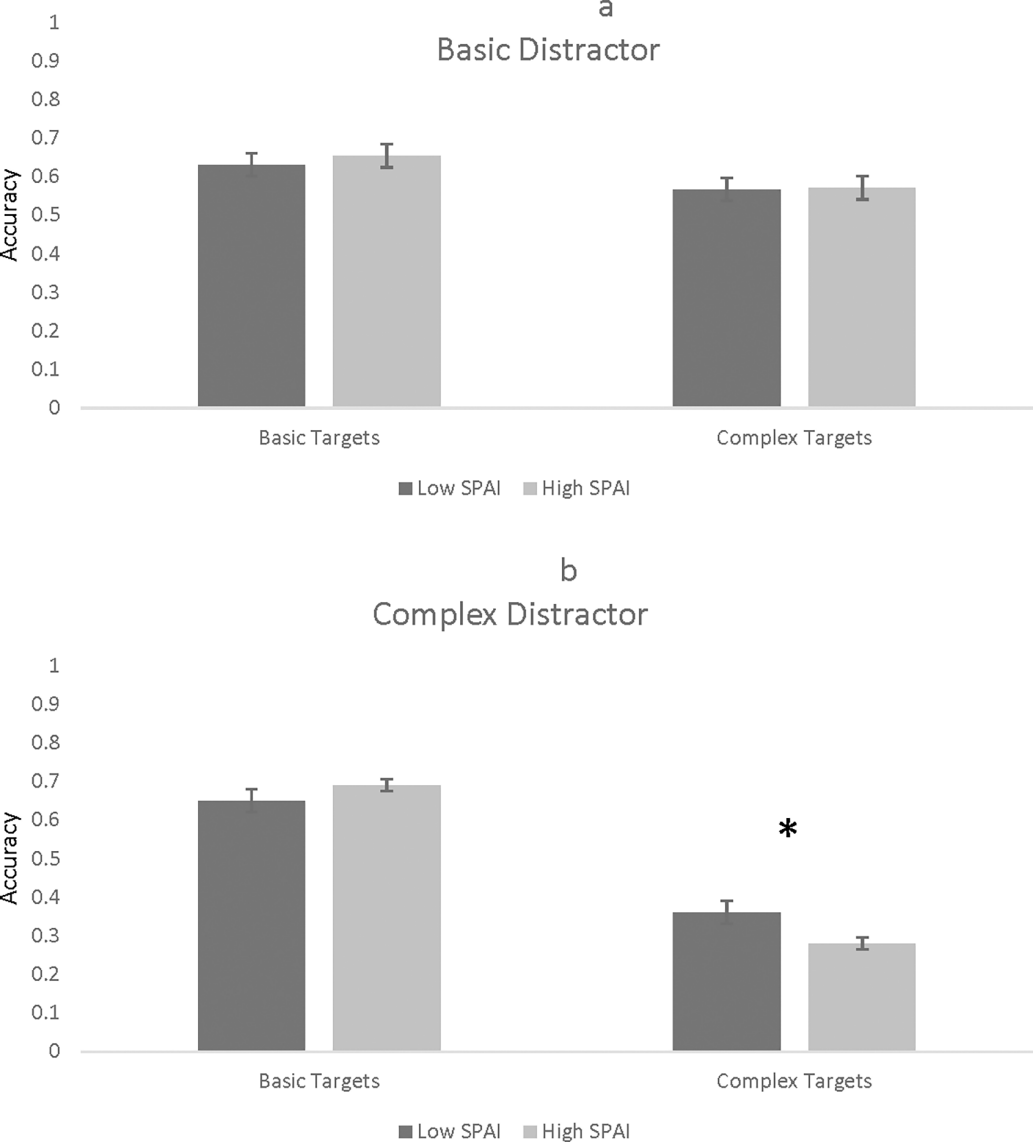
Accuracy as a function of flanker complexity, target complexity, and SPAI for trials with 16 faces. Fig 5a depicts basic distractors while Fig 5b shows complex distractors. The asterisk indicates statistical significance at *p* < .05. Error bars represent standard errors.

A 2 (Number of Stimuli: 8, 16) x 2 (Target: basic, complex) x 2 (Distractor: basic, complex) x 2 (SPAI Group: high, low) mixed factor ANOVA revealed a Number of Stimuli x Target x Distractor x SPAI interaction, *F*(1, 112) = 5.67, *p* = .019, *η*_*p*_^*2*^ = .048. To investigate this interaction, Target x Distractor x SPAI mixed model ANOVAs were conducted separately for each Number of Stimuli condition. For trials with 8 faces, there was not a Target x Distractor x SPAI effect, *F*(1, 112) = 0.36, *p* = .455, *η*_*p*_^*2*^ = .005. For trials with 16 faces, however, there was a statistically significant Target x Distractor x SPAI effect, *F*(1, 112) = 9.23, *p* = .003, *η*_*p*_^*2*^ = .076. This interaction was further broken down by distractor type. For basic distractors, there was not a Target x SPAI interaction, *F*(1, 112) = 0.42, *p* = .519, *η*_*p*_^*2*^ = .003 (see Fig 5A). For complex distractors, however, there was a significant Target x SPAI interaction, *F*(1, 112) = 6.39, *p* = .013, *η*_*p*_^*2*^ = .054 (see Fig 5B). Finally, simple main effects analyses were conducted. Results indicated that for complex targets, *F*(1, 112) = 3.80, *p* = .054, *η*_*p*_^*2*^ = .033, high SPAI participants were less accurate than low SPAI participants. High SPAI and low SPAI participants did not differ in their accuracy for basic targets, *F*(1, 112) = 1.99, *p* = .161, *η*_*p*_^*2*^ = .017.

#### Emotion valence

**Fig 6:**
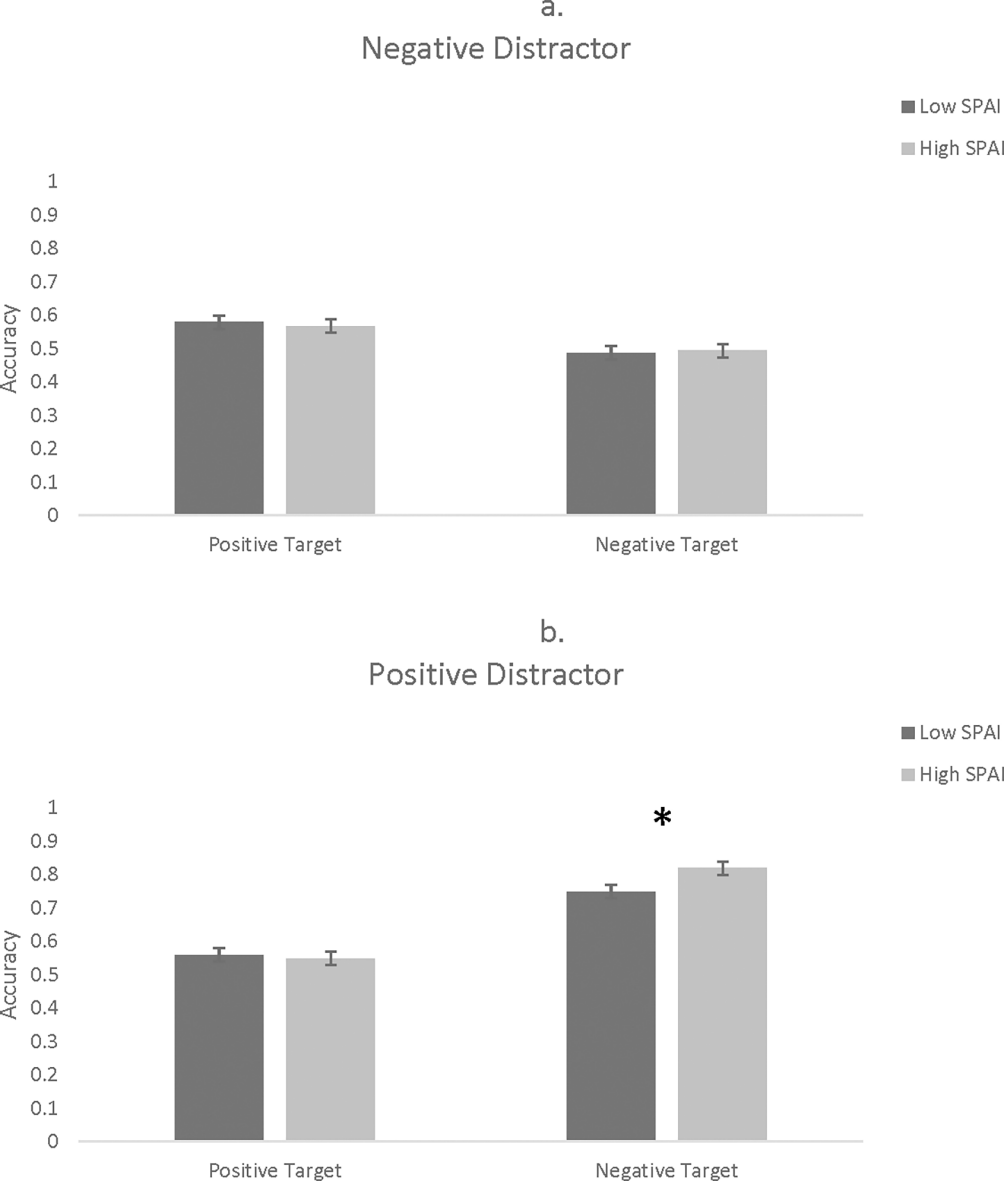
Accuracy as a function of flanker valence, target valence, and SPAI. Fig 6a depicts negative distractors while Fig 6b shows positive distractors. Asterisks indicates statistical significance at *p* < .05. Error bars represent standard errors.

A 2 (Number of Stimuli: 8, 16) x 2 (Target: positive, negative) x 2 (Distractor: positive, negative) x 2 (SPAI Group: high, low) mixed factor ANOVA revealed a Target x Distractor x SPAI interaction, *F*(1, 112) = 11.31, *p* = .001, *η*_*p*_^*2*^ = .092. This effect was broken down first by distractor type. When distractors were negative, the Target x SPAI interaction was not significant, *F*(1, 112) = 0.55, *p* = .458, *η*_*p*_^*2*^ = .005 (see Fig 6A). When distractors were positive, however, there was a Target x SPAI interaction, *F*(1, 112) = 6.07, *p* = .015, *η*_*p*_^*2*^ = .051 (see Fig 6B). Simple main effects analyses revealed that, with positive distractors, the high SPAI group did not differ from the low SPAI group, *t*(112) = 0.22, *p* = .827. However, when the targets were negative, the high SPAI group was more accurate than the low SPAI group, *t*(112) = -2.00, *p* = .048.

## Discussion

The current research investigated the unique contributions that traits related to autism and social anxiety have on social cognitive tasks involving emotional faces. We examined typically-developing individuals who varied along the BAP. This approach allowed us to investigate mechanisms that may underlie ASD and assess challenges faced by individuals with high numbers of autistic traits. Study 1 assessed emotion identification in which task performance in the form of reaction time was related to the control of attention away from distracting stimuli. As expected, participants high in autistic traits performed more slowly on this task than those low in autistic traits when stimuli depicted complex emotions. Study 2 examined the processing of a small or a large number of emotional faces in which attention to detail, a strength associated with autism, was necessary for successful task performance as assessed by accuracy. Perhaps as a result of attention to detail skills, participants with autistic traits performed equally well on all types of trials. Performance on this task, as hypothesized, differed as a function of social anxiety, which is consistent with previous research showing different levels of attention to emotional faces varying by emotion type. Thus, our findings show differences in processing emotional faces between those high or low in autistic traits compared with those who are high and low in social anxiety. Compared to those low in autistic behaviors, those high in autistic behaviors tend to focus on a detailed stimulus, but are affected negatively by distracting stimuli. Those with high social anxiety are disrupted when a large number of emotional faces are presented.

In Study 1, reaction times during emotion identification were slower during the most cognitively taxing conditions for participants high in autistic traits, when both the target and flanker included complex emotions. The present work is novel in that it demonstrates that the processing of complex emotions may require additional cognitive resources for these individuals, which may detract from their ability to perform other tasks [67]. This finding has implications for a number of social situations. For example, in a face-to-face conversation in which effective communication involves the evaluation of another’s emotional expressions, individuals with high numbers of autistic traits may be at a disadvantage when trying to interpret complex emotional expressions. These findings support the idea that autistic-like behaviors occur along a continuum and highlight the importance of examining the impact of these behaviors in situations that jointly tax emotion processing and attention. In Study 2, the performance of individuals with autistic traits was not impaired on a task that involved both emotion identification and attention to detail, most likely because of the superior attention to detail skills associated with autism, which could be used to discriminate between small details in different faces. Indeed, both high and low AQ participants were more accurate when there were 8 stimuli than 16 stimuli, demonstrating the same pattern of responses when the number of stimuli were varied. Furthermore, responses based on emotion complexity did not differ as a function of AQ. The ability of individuals with autistic traits to fixate on specific facial features such as the mouth rather than processing the holistic facial expression (e.g., [10,12]), may have assisted them in this task.

Whereas individuals with varying levels of traits related to social anxiety did not perform differently on the flanker task in Study 1, performance instead varied on the visual search task in Study 2. High SPAI participants performed less accurately on trials showing all complex faces. This is consistent with previous research suggesting that social anxiety is related to a deficit in recognizing complex versus basic emotions [49]. In the present study, this result was only found on trials depicting a large number of faces (i.e., 16). This result is in line with other research demonstrating that socially anxious individuals view emotional faces as threatening, which may lead to an attentional bias away from faces, particularly those they are already poor at identifying (i.e., complex emotions; [61]). Study 2 also revealed that participants with higher SPAI scores were more accurate than participants with lower SPAI scores when the target faces were negative and the distractors were positive. This result is consistent with previous work showing that socially anxious individuals show a deeper neural processing of negative versus positive emotional faces [50] which could lead these individuals to attend more to negative than positive emotions. Taken together, these results demonstrate that participants high in traits related to social anxiety show different patterns of responses to emotional faces varying in emotion complexity and emotion valence. Different patterns of attention could have implications for socially anxious individuals in social situations. For example, when there is a group of people with which the individual is interacting, socially anxious individuals may be less able to recognize complex emotions, which may impair social skills in this setting but may be particularly adept at attending to and recognizing negative faces in this context. This may be an advantage in some cases in which recognizing the fearful or angry person could be important or a disadvantage in other cases in which attention is drawn away from the majority of people in the social situation to focus on one individual.

There are several limitations of this work that should inform future research. One limitation is our focus on college students as participants. We chose this sample because the university setting has been shown to be challenging for individuals with ASD [68,69] and social anxiety but is often under-diagnosed (e.g., [70,71]). Future work should test to see whether the findings reported herein generalize to other BAP populations. Another limitation of our study is the use of static stimuli to assess emotion identification. As emotions are conveyed in real social situations with dynamic movements, these findings may not generalize to everyday social situations. Future research should use dynamic faces to assess emotion and attention processing in these groups. Finally, it is important to note that many of the effect sizes are relatively small. Small effect sizes, perhaps, could be expected when studying the BAP, given that there is considerable heterogeneity in task performance in those diagnosed with ASD [26]. Another future research direction is to further study the different forms of variance that can impact task performance in individuals along the BAP.

In sum, performance on two social cognitive tasks differed as a function of both the task as well as individual traits associated with autism and social anxiety. In a task that involved the identification of emotions and the control of attention, participants with more autistic traits showed impairments in trials with complex emotions but performance was unaffected by traits related to social anxiety. On the task that involved emotion identification and attention to detail, participants high in traits related to social anxiety were less accurate at identifying complex emotions when there was a large number of faces presented and more accurate when target faces depicting negative emotions. Our work demonstrates that individuals on the BAP show both strengths and weaknesses associated with autistic traits and social anxiety that may have implications for social situations. One future direction would be to understand whether there are differences in neural signatures or other physiological measures between those high and low in autistic traits compared with those high and low in social anxiety that could be used to predict differences in task performance in these groups. Another research direction, given the variability in our results, would be to better understand factors that protect those with high autistic traits or high social anxiety traits from group differences in facial emotion processing that we found in the present studies. Understanding the unique contributions of these traits in different tasks that necessitate a different skill set can help inform training or interventions for BAP individuals and can also contribute to our understanding of ASD. Based on our present findings, trainings that strengthen executive functioning, specifically the ability to focus on a face emotion with distracting stimuli present, may be a useful strategy to improve emotion recognition.

## Supporting information

S1 File SPSS data file for Study 1. (SAV)

S2 File SPSS data file for Study 2. (SAV)
